# Biogenic Composite Filaments Based on Polylactide and Diatomaceous Earth for 3D Printing

**DOI:** 10.3390/ma13204632

**Published:** 2020-10-16

**Authors:** Marta Dobrosielska, Robert Edward Przekop, Bogna Sztorch, Dariusz Brząkalski, Izabela Zgłobicka, Magdalena Łępicka, Romuald Dobosz, Krzysztof Jan Kurzydłowski

**Affiliations:** 1 Faculty of Materials Science and Engineering, Warsaw University of Technology, 141 Wołoska, 02-507 Warsaw, Poland; marta.dobrosielska@pw.edu.pl (M.D.); romuald.dobosz@pw.edu.pl (R.D.); 2 Centre for Advanced Technologies, Adam Mickiewicz University in Poznań, 10 Uniwersytetu Poznańskiego, 61-614 Poznań, Poland; bs33013@amu.edu.pl; 3 Faculty of Chemistry, Adam Mickiewicz University in Poznań, 8 Uniwersytetu Poznańskiego, 61-614 Poznań, Poland; db85077@amu.edu.pl; 4 Faculty of Mechanical Engineering, Bialystok University of Technology, 45C Wiejska, 15-351 Bialystok, Poland; i.zglobicka@pb.edu.pl (I.Z.); m.lepicka@pb.edu.pl (M.Ł.); krzysztof.kurzydlowski@pw.edu.pl (K.J.K.)

**Keywords:** diatoms, fused deposition modeling (FDM), polylactide, 3D printing

## Abstract

New composites containing a natural filler made of diatom shells (frustules), permitting the modification of polylactide matrix, were produced by Fused Deposition Modelling (3D printing) and were thoroughly examined. Two mesh fractions of the filler were used, one of <40 µm and the other of 40−63 µm, in order to check the effect of the filler particle size on the composite properties. The composites obtained contained diatom shells in the concentrations from 0% to 5% wt. (0−27.5% vol.) and were subjected to rheological analysis. The composites obtained as filaments of 1.75 mm in diameter were used for 3D printing. The printed samples were characterized as to hydrophilic–hydrophobic, thermal and mechanical properties. The functional parameters of the printed objects, e.g., mechanical characteristics, stability on contact with water and water contact angle, were measured. The results revealed differences in the processing behavior of the samples as well as the effect of secondary granulation of the filler on the parameters of the printing and mechanical properties of the composites.

## 1. Introduction

Additive technologies, known also as 3D printing, have become a key type of processing of 4.0 Industry. They were developed in the beginning of the 1980s, however their rapid growth started nearly a decade ago, mainly because of finding solutions permitting cost reduction, and cessation of patent protection. Until that time, these technologies were inaccessible for individuals, small companies, research and technology workers concerned with the improvement of technologies and designing new materials. At present, the area of additive technologies has entered a new phase of development and arouses increasing interest in many branches of the industry. A wide range of materials can be applied in this technology, starting from plastics, through ceramics to metal powders. For each type of material, different dedicated techniques and types of 3D printers are used. Fused Deposition Modeling (FDM) is a popular method for the production of elements made of thermoplastic materials [1,2].

The widespread use of FDM is strictly related to the development of printing materials and 3D printers. Additive technologies are used, e.g., in medicine for printing polymer implants, showing high density and rigidity, which can remain in human body for a longer time than traditionally produced ones [3]. The products of 3D printing find increasing use in the automotive and aviation industry and architecture [4]. The most important challenges in the research and development of FDM include the improvement of mechanical strength and functional properties of the products at a high rate of the printing process. One of the materials most often used for 3D printing is polylactide (PLA), obtained from renewable and biodegradable precursors [5,6]. According to IUPAC (International Union of Pure and Applied Chemistry), its proper simplified name is poly(lactide), however it has been often ambiguously referred to as poly(lactic acid), despite not containing carboxyl groups [7]. The greatest benefits of PLA in the area of material processing is its ability to biodegrade in specific conditions, low glass transition point, small thermal shrinkage upon extrusion molding (also in 3D printing) and no toxic gases emission upon processing. Polylactide is not counted as a good construction material as it shows a low impact strength and low thermal stability [8]. It is used for the production of everyday use goods, including the packaging foil, disposable products and containers, and medical products such as wound dressings, threads and surgical masks [9]. To not compromise the ecologically friendly character of the products, PLA, which is a biopolymer, should be used with additives of natural origin. One of possible additives can be diatom shells made of biosilica. Diatoms are one of the most abundant and diverse groups of algae. Their characteristic feature is the decorated silica shells (frustules) of a variety of shapes and sizes from 1 μm to 1 mm, most often 10–200 μm [10]. Due to their size, diatoms belong to microorganisms. Thanks to their abundant presence in water, the bottoms of all kinds of water bodies are covered with diatomaceous earth formed of diatom shells [11]. Fossil diatoms are found in the form of diatomite or diatomaceous earth. The content of silica in diatomite varies from 60% to 95%, the rest are pollutants such as carbonates, iron oxides, quartz, loams or substances of volcanic origin. One cm^3^ of diatomite contains about 2.5 billion of diatom shells [12,13]. Thanks to its attractive properties, e.g., high sorption capacity, abrasive character, insecticide activity and nontoxicity, it is a valuable component used in food and chemical industries, and construction. Diatom shells are covered with a variety of pore systems, different for individual species, but also different in the shell of each diatom. The pores of different shapes and sizes permit the capturing of nutrients and filtering off toxic substances. Diatoms are a renewable source of three-dimensional nanostructural silica that could be used in different devices filtering pathogens (bacteria or viruses). As diatomite shows the ability to adsorb heavy metal ions (nickel, lead, zinc, and titanium) and participate in water oxidation, it is used for water treatment [14,15]. Moreover, diatomite is used for purification and filtration of alcohols, saccharides, paints and varnishes, as well as for protection and thermal insulation. The first report on the application of PLA filaments enriched in diatom shells in FDM has been presented by Aggarwal et al. [16]. From the point of view of processing, the important parameters describing the diatomaceous earth as a filler are the primary and secondary granulation. The primary granulation is the actual size of the diatom shells used in the filler, while the secondary granulation is the size of agglomerated and non-agglomerated diatom shells in the composite filaments. A distinction between these two types of granulation is vital from the point of view of processing and the properties of the composite materials based on diatomaceous earth. Besides the specific properties of diatoms, attractive for 3D printing when in composite with PLA, the application of a biogenic filler can considerably reduce the cost of this process. In this work, we present an approach towards the preparation of composites comprised of PLA as a polymer matrix and diatomite as an extender and structural filler, allowing for an improvement to the mechanical characteristics of PLA and a reduction in the polylactide consumption. The obtained materials were formed into 3D printing filaments and showed satisfactory printability, which was proof of the concept for a new series of highly eco-friendly, biodegradable and multi-purpose materials for the FDM technique. These composite filaments may be exceptionally attractive for the introduction to consumer use due to the amount of globally produced plastic waste of household−3D printing origin, which is often not properly disposed of and usually not recovered for recycling.

## 2. Materials and Methods

### 2.1. Materials

Polylactide (PLA) type Ingeo 2003D was purchased from NatureWorks (Minnetonka, MN, USA). Diatomaceous earth—fossilized algae diatoms of Phylum Bacillariophyta from pure Mexican fresh water was purchased from Perma Guard Agro (Otwock, Poland).

### 2.2. Analyses

Contact angle analyses were performed by the sessile drop technique at room temperature and atmospheric pressure, with a Krüss DSA100 goniometer (KRÜSS GmbH Hamburg, Germany). Three independent measurements were performed for each sample, each with a 5 µL water drop, and the obtained results were averaged to reduce the impact of surface nonuniformity.

Thermogravimetry was performed using a NETZSCH 209 F1 Libra gravimetric analyzer. (NETZSCH-Gerätebau GmbH, Selb, Germany). Samples of 5 ± 0.2 mg were cut from each granulate and placed in Al_2_O_3_ crucibles. Measurements were conducted under nitrogen (flow of 20 mL/min) in the range of 30–800 °C and a 20 °C/min heating rate. Differential scanning calorimetry was performed using a NETZSCH 204 F1 Phoenix calorimeter (NETZSCH-Gerätebau GmbH, Selb, Germany). Samples of 6 ± 0.2 mg were cut from each granulate and placed in an aluminum crucible with a punctured lid. The measurements were performed under nitrogen in the temperature range of −20–290°C and at a 20 °C/min heating rate, and T_g_ was measured from the second heating cycle.

The effect of the modifier addition on the mass flow rate (MFR) was also determined. The measurements were made using a Instron plastometer, model Ceast MF20 (Instron, Norwood, MA, USA) according to the applicable standard ISO 1133. The measurement temperature was 190 ± 0.5°C, while the piston loading was 2.16 kg.

The dynamic viscosity coefficient was determined by a capillary rheometer Instron Ceast SR 10 (Instron, Norwood, MA, USA), according to ISO 11443:2005, using a capillary tube of 5 mm in length, 1 mm in diameter and the shearing speed range 1−100 (s^−1^). Measurements were carried out at 190 °C.

For flexural and tensile strength tests, the obtained materials were printed into type 1B dumbbell specimens in accordance with EN ISO 527:2012 and EN ISO 178:2006. Tests of the obtained specimens were performed on a universal testing machine INSTRON 5969 with a maximum load force of 50 kN. The traverse speed for tensile strength measurements was set at 2 mm/min, and for flexural strength was also set at 2 mm/min. Charpy impact test (with no notch) was performed on a Instron Ceast 9050 (Instron, Norwood, MA, USA) impact-machine according to ISO 179−1. For all the series, 6 measurements were performed.

The X-ray diffraction measurements were carried out using a Philips PW1050 diffractometer (Philips, Amsterdam, The Netherlands) working in the θ−2θ geometry with Ni-filtered CuKα radiation. The following measurement conditions were applied: 2θ range of 5°−80°, voltage 35 kV, current 20 mA, scan step 0.040° at 1° per minute. The positions of the reflections were calculated by the Philips APD program version 1.3.

The porous structure was determined by low temperature nitrogen adsorption measurements carried out on a Micromeritics ASAP 2420 apparatus (Norcross, GA, USA) in the standard analysis mode, using 0.8−1.4 g of material with the grain size fraction between 0.1 and 0.2 mm. Prior to nitrogen adsorption, all samples were degassed for about 10 h at 350 °C at 0.4 Pa until reaching a constant weight. Both adsorptive and desorptive branches of the isotherm were recorded in the range of p/p_0_ 0−1.0. Reports were provided by ASAP 2420 software (Micromeritics Instrument Corp., version 2.09A). Distribution of pore area and pore volume was calculated using de Boer t-plot method and BJH method. The pore volume and pore diameter were established from the adsorptive branch of the isotherm using BJH method, and the surface area was calculated using the BET method.

Hardness of the composite samples was tested by the Shore method using a durometer Bareiss Prüfgerätebau GmbH (Oberdischingen, Germany).

The images of diatomaceous earth as well as composite materials were obtained using a scanning electron microscope Hitachi SU−8000 (Tokyo, Japan) using acceleration voltage of 5.0 kV. Samples were placed on the double-adhesive carbon tape and coated with Cu-Ni, using the PECS coating system made by Gatan (Pleasanton, CA, USA).

The grain size distribution was measured by a Mastersizer 3000 (Malvern Instruments Ltd. Malvern, UK). The measurements were made for the samples in the water suspension (Hydro EV attachment) and for dry powders (Aero S attachment). The parameters of measurements for dry powders: rate of sample supply 27, air pressure 2.7 bar, and nozzle slit 1 mm. The parameters of measurements for wet samples: stirrer revolution speed 2330 rev/min and ultrasound power 60%.

A water absorption study of printed samples was also carried out. The samples were placed in distilled water at 50 °C upon constant stirring with a magnetic stirrer for 72 h. The samples were then pre-dried, placed under vacuum for 1 h, and then in an oven at 40 °C for 24 h.

### 2.3. Fabrication of Filaments

The filaments were fabricated using the following procedure: (a) preparation of diatom modifier, (b) preparation of concentrated granules, and (c) extrusion of filaments.

(a) Preparation of diatom modifier

Diatomaceous earth was milled in a ball mill and then divided into fractions (on a vibrating table) of the mesh size up to 40 µm and 40−63 µm to be used in further studies.

(b) Preparation of granulates

The polymer and the filler were homogenized using a laboratory two-roll mill ZAMAK MERCATOR WG 150/280 (Krakow, Poland). A portion of 500 g PLA Ingeo™ 2003 D was mixed with 26.5 g diatomite of grain size up to <40 µm or from the range 63−40 µm, until the final concentration of the filler of 5% *w*/*w*. The mixing was performed at the rolls temperature of 200 °C for 15 min., getting to full homogeneity of the concentrates. Masterbatch was granulated by a grinding mill WANNER C17.26 sv. (Wanner Technik GmbH, Reicholzheim, Germany) The granulates were diluted with pure PLA up to the final filler concentrations of 1% or 2.5% *w*/*w* upon extrusion molding of a stream with consequent cold granulation on the twin-screw extrusion setup line HAAKE Rheomex OS (Thermo Fisher Scientific, Waltham, MA, USA), and then dried for 24 h at 40 °C.

(c) Extrusion of Filaments

The granulates obtained as above were used for molding of filaments of 1.75 mm in diameter by a single-screw extrusion setup HAAKE Rheomex OS (Thermo Fisher Scientific, Waltham, MA, USA).

#### 3D Printing (FDM)

Using a 3D printer FlashForge Finder (Warsaw, Poland) two types of samples were printed by FDM: oars and bars, according to PN-EN- ISO 527−2. Parameters of printing are given in Table 1.

## 3. Results and Discussion

### 3.1. Low-Temperature Nitrogen Sorption

**Figure 1:**
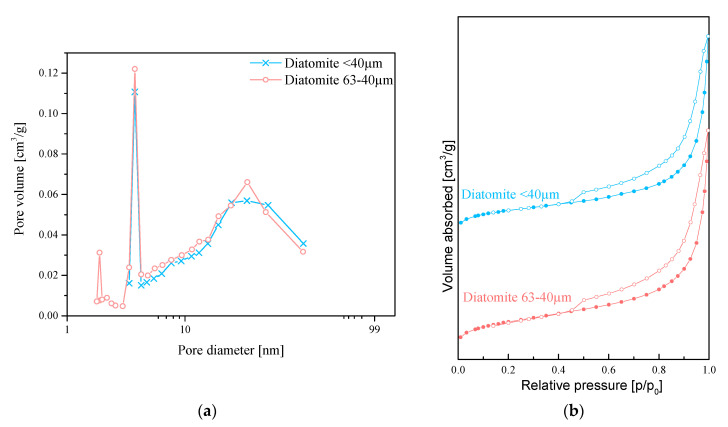
(**a**) Pore volume versus diameter and (**b**) isotherms of nitrogen sorption.

Diatomaceous earth has a triple hierarchical structure as it shows three types of pore systems: micropores of 2−3 nm, mesopores of 10−50 nm and macropores of 3−10 μm in diameter. Thus, diatom shells are nanoporous materials of hierarchic porous structure [17]. Specific surface area of diatomite varies depending on its origin. The mean volume of pores obtained from the desorption branch of nitrogen sorption was 0.063 cm^3^/g and 0.070 cm^3^/g for the fractions of <40 µm and 63−40 µm, respectively. The mean pore diameter obtained by the same method was 13.51 nm and 11.70 nm, which confirm the presence of small mesopores in the frustule. The specific surface area of the fractions was 24.40 ± 0.18 m^2^/g for the diatomite fraction of <40 µm, while 27.60 ± 0.16 m^2^/g for the fraction of 63−40 µm, respectively. The results are given in Figure 1a,b. The specific surface areas of other fillers vary in a wide range, e.g., the specific surface areas of aluminum oxide (Al_2_ O_3_), silica (SiO_2_), magnetite (Fe_3_ O_4_), zinc oxide (ZnO), magnesium hydroxide (Mg(OH)_2_) are 12−250 m^2^/g [18], 50−1000 m^2^/g [19], 6−90 m^2^/g [20], 8−75 m^2^/g [21,22], and 4−10 m^2^/g [23], respectively. The surface area of the diatomite is not as well-developed as that of, e.g., aluminum oxide, but it still has been successfully used as an adsorbent thanks to its porous structure [24].

### 3.2. Particle Size Measurements by Dynamic Light Scattering (DLS)

**Figure 2:**
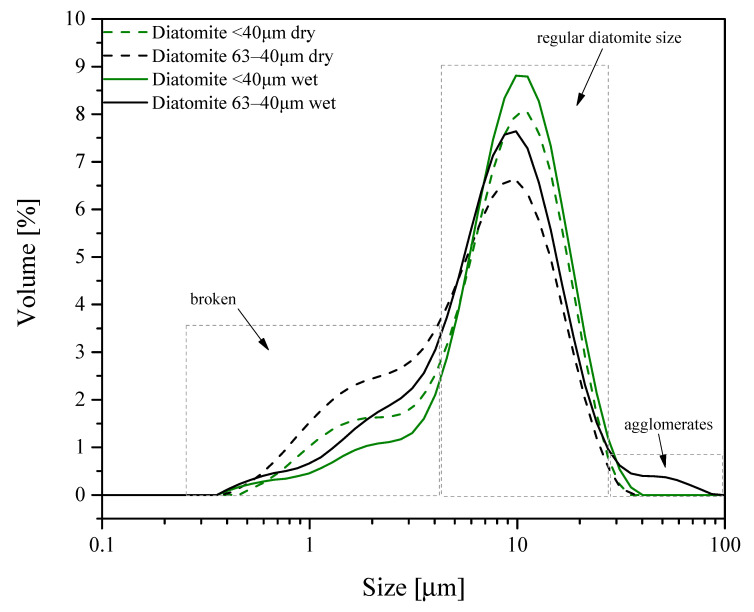
Particle size distribution of diatom shells for the two mesh size fractions; measurements in water and in dry powder.

**Figure 3:**
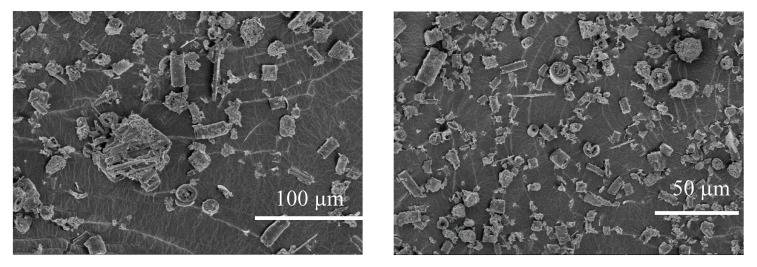
SEM image of dry powder of amorphous diatomaceous earth.

Amorphous diatomaceous earth was sieved into two mesh size fractions: one of grans smaller than 40 µm and the other of grain size 40−63 µm, in order to elucidate the problem of formation of diatom shells agglomerates in the diatomaceous earth. Figure 2 shows many agglomerates of diatom shells whose appearance may imply poorer dispersion of the filler in the PLA matrix. Determination of the size of particles in dry powders and in water suspension in the two size fractions (Figure 2) revealed differences in particle distribution in them. The fraction of particles size 63−40 μm shows a greater contribution of particles smaller than 6 μm, classified on the basis of SEM images as fragments of broken diatom shells (cf. 3.3 SEM). This fraction contains a smaller number of the largest particles of sizes close to 40 μm. Both mesh fractions contain the greatest number of particles of sizes close to ~10 μm (the fraction <40 µm contains about 15% more). Measurements performed in water permitted further observations. The fraction <40 µm after immersion in water showed a minimum increase in the maximum particle size, which was still below 40 μm, although their contribution significantly increased. The fraction 40−63 µm shows the appearance of a small contribution of particles whose size reaches up to 90 μm. This observation indicates the agglomeration of diatom shell particles in water. The diversity of particle sizes in both mesh fractions 40−63 µm and <40 µm is visible in the corresponding SEM images shown in Figure 3.

The DLS method was chosen to gain information on the actual behavior of the particles. The measurements for dry powders provides more reliable particle sizes, and thanks to the way the samples were supplied, there is no problem with particle agglomeration. The particle size profile obtained from such measurements corresponds to that of the primary particles. The measurements in water do not eliminate the problem of particle agglomeration (as seen in the particle size profile), but are useful for the estimation of the actual secondary particle size in composite systems in which the filler of polar surface shows a tendency to agglomerate in a much less polar polymer matrix. Thus, to get a wider picture of particle size distribution, the two methods have to be employed side-by-side.

### 3.3. SEM

**Figure 4:**
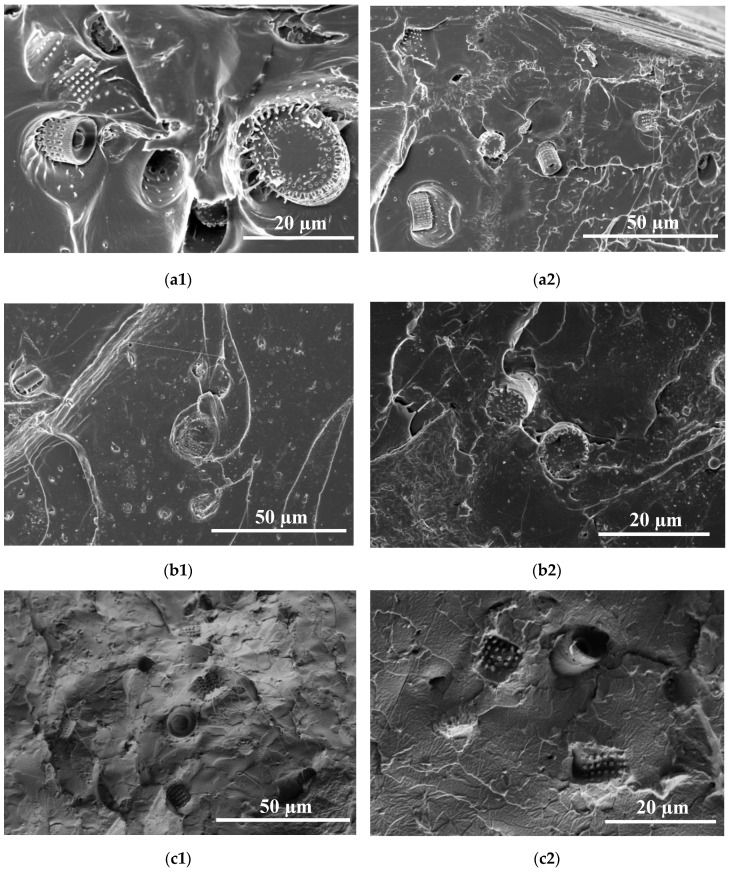

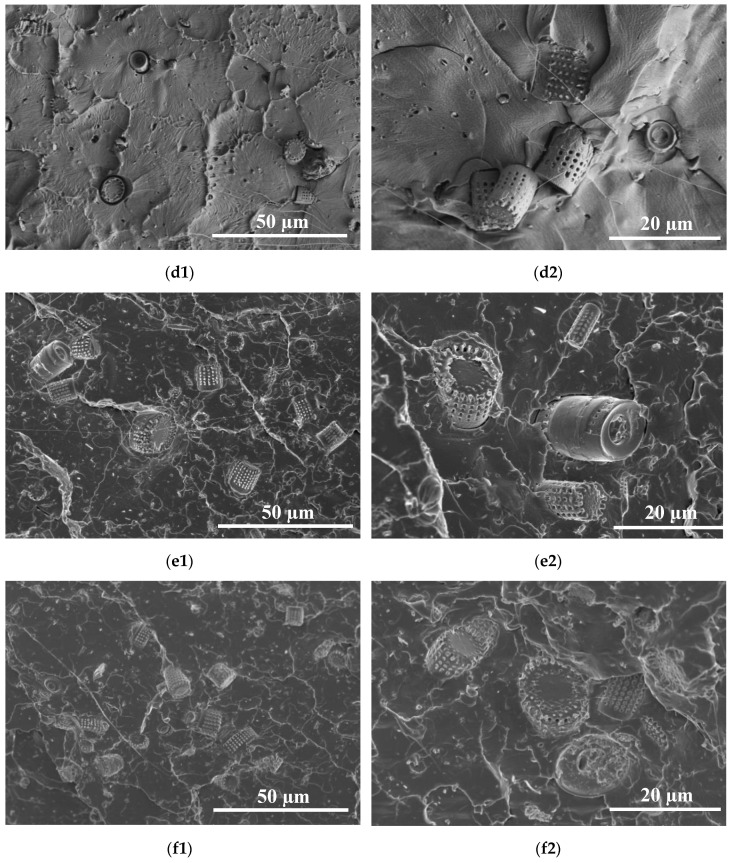
SEM images of PLA composites with diatoms: (**a1**) 1% diatomite fraction <40 µm, scale 50 µm, (**a2**) 1% diatomite fraction <40 µm, scale 20 µm, (**b1**) 1% diatomite fraction 63−40 µm, scale 50 µm, (**b2**) 1% diatomite fraction 63−40 µm, scale 20 µm, (**c1**) 2.5% diatomite fraction <40 µm, scale 50 µm, (**c2**) 2.5 % diatomite fraction <40 µm, scale 20 µm, (**d1**) 2.5 % diatomite fraction 63−40 µm, scale 50 µm, (**d2**) 2.5 % diatomite fraction 63−40 µm, scale 20 µm, (**e1**) 5% diatomite fraction <40 µm, scale 50 µm, (**e2**) 5% diatomite fraction <40 µm, scale 20 µm, (**f1**) 5% diatomite fraction 63−40 µm, scale 50 µm, and (**f2**) 5% diatomite fraction 63−40 µm, scale 20 µm.

The SEM imaging of the samples broke the cross sections and revealed the presence of diatom shells in the PLA matrix and permitted the evaluation of the size, morphologies and defects of the filler particles, as well as their distribution and agglomerations (Figure 4a–f). The figures show diatom shells of different shapes and sizes. SEM images of raw fossil diatomaceous earth showed that it contained unbroken diatom shells of a cylindrical shape of 8 to 20 μm in diameter, along with broken shells of sizes below 8 μm and shell agglomerates of 20 to 80 μm in size. With increasing concentrations of diatoms in the matrix, their agglomerates become larger, irrespective of the fraction. The majority of the diatom shells were visible in the SEM images and assumed the shape of a cylinder and had numerous openings; only few are built of smooth walls. This characteristic feature of diatom shells permitted living diatoms to absorb nutrients and excrete products of metabolism. In the context of their effect on the properties of diatom shells as a filler, these openings can be treated as pores. The pores play an important role in the process of the filler binding with the polymer matrix, which has been confirmed by SEM image analyses.

**Figure 5:**
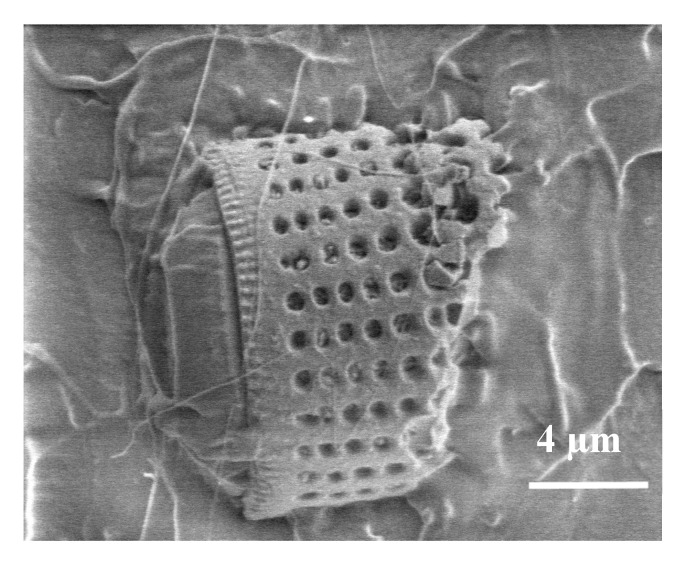
SEM image of the sample of PLA with 1% *w*/*w* loading of diatomite, fraction <40 µm.

Figure 4 presents images of composites of PLA with diatomites of different particle size fractions and added in different concentrations. The images reveal distinct non-agglomerated diatom shells, usually of cylindrical shape. They have different diameters, varying from about 7 µm to 20 µm, however those of the external diameter of approx. 15 µm and 9−12 µm in length dominate. The shells are covered with clearly marked pores of 0.5−0.6 µm in diameter (Figure 5).

**Figure 6:**
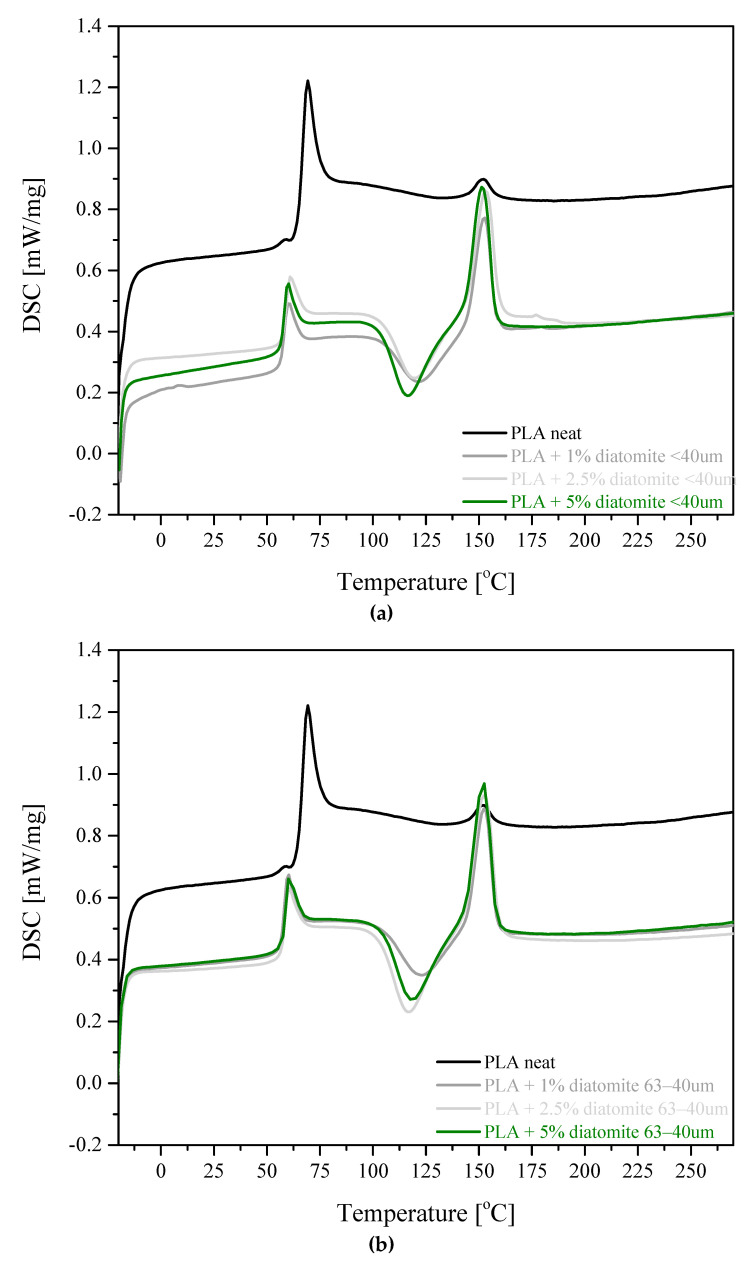
DSC curves recorded for samples of PLA composite with diatomite (**a**) <40 µm and (**b**) 63−40 µm.

Usually, the well-defined oval shape pores make a network. As a result of the processing, the secondary particles underwent de-agglomeration, which points to good dispersion of the diatom shells in the PLA matrix and high miscibility of the filler with the matrix. The images also show the traces of polymer flow through the pores in diatom shells, Figure 6a, as trails of PLA running from the pore mouth to the bulk polymer. This observation indicates a very good penetration of frustules by the molten polymer and good mechanical contact of the filler with the matrix, which means that this filler has an effective reinforcing phase.

### 3.4. DSC and TGA Results

Results of the TGA and DSC measurements, in the form of DSC curves, are presented in Figure 6a,b, and the parameters determined, including the percentage of the mass loss, temperature at the maximum rate of mass loss and glass transition temperature are given in Table 2 and Table 3. The processes of decomposition of the reference sample and composites of PLA with diatom shells occur in a single stage. Thermal decomposition of both fractions of the filler in the form of dry powder takes place in three stages. The first one in the range of 228.8−234.8 °C is interpreted as the evaporation of water physically and chemically adsorbed in the pores of diatom shells. The mass loss accompanying the process is 8−9%. The second mass loss is observed in the range 450.7−457.5 °C is interpreted as corresponding to the decomposition of organic and inorganic substances as well as the elimination of remaining silanol groups. The third stage was noted to take place in the range of 962.9−983.5 °C could be explained by the total dehydration of the sample and decomposition of carbonates [25]. On the basis of the derivatographic curves, it was possible to establish the temperature of thermal decomposition (pyrolysis) of molten mixtures of PLA and diatomaceous earth. On the basis of T_onset_ it can be concluded that in the first phase of the samples decomposition, the presence of diatom shells has a stabilizing effect on the matrix, for instance through hindering of free radicals transfer between the polymer chains and slower radical migration in more viscous polymer melt. The sample of pure PLA was characterized by the lowest T_onset_ from among all samples, of 348.3 °C. However, when comparing the temperatures of the fastest mass loss (T_DTG_), the sample of pure PLA showed the highest from among all the samples, at 376.0 °C. In these conditions, diatom shells acted as catalyst of thermal decomposition, similarly as zeolites used in the catalytic pyrolysis of organic matter, including polymers. The decrease in T_DTG_ being rather small was related to a low contribution of diatom shell mass in the composites [26]. The dry mass left after pyrolysis increased, as expected, with a growing concentration of the filler and with an increasing size of the filler fraction used. The differences between the dry mass left after pyrolysis (Table 2) and the actual content of the filler in the composite follow from the fact that the process of pyrolysis is rapid (a steep mass loss curve) and pyrolytic gases carry out some fragments of diatom shells [27].

The glass transition temperature (T_g_), determined on the basis of the second cycle of heating is clearly lower for all the samples containing diatom shells, than for the reference sample (59.9 °C, Table 3). It is most probably related to the presence of additional stress in the sample detected by the XRD method and the presence of additional amorphous interphases at the PLA-filler interface or to a negative influence of the filler on the orientation ability of PLA chains. Exothermic peak appearing on the heating curve at about ~120 °C is assigned to “cold crystallization”, T_cc_. In this process a small amount of the polymer undergoes crystallization upon heating [28]. The decrease in T_cc_ is interpreted as due to the presence of internal stress in the sample. An important parameter of sample processing is the time of the cycle, which should be the shortest possible, to maximize the efficiency of production. For this reason the process of cooling is fast and short lasting, which is not conducive to crystal phase development. The melting point of the PLA crystalline fraction is in the range of 150−154 °C, the signals area was higher for neat PLA than for the samples with the filler (Table 3, k +5%a–b). This observation can be explained by the fact that diatom shells may act as the nuclei of crystallization and boosts the formation of crystallites [29]. An addition of diatomaceous earth to the polymer significantly reduced the contribution of the amorphous fraction and increased that of the crystalline phase. Moreover, with increasing content of diatom shells the crystallization point was noted to decrease. No significant effect of the filler addition on the melting point of the composites was observed.

### 3.5. Contact Angle Measurements

Water contact angle (WCA) was measured for dry samples and the samples subjected to distilled water at 50 °C (chemical treatment). Results of the measurements are given in Table 4. Diatomaceous earth is a hydrophilic filler, characterized by a water contact angle of 0°. The degree of hydrophobicity decreases with the increasing particle size of the diatom shells. The samples containing diatomite of particle size fraction 63−40 µm are characterized by a smaller water contact angle (below 90°) than neat PLA (93.5°) so are hydrophilic. However, the composites of 40 µm particle fraction are more hydrophobic than the neat PLA. This difference between the WCA of two mesh fraction composite series is the result of the difference in the particle size of these two fractions, which in turn introduces additional porosity to the sample surface in the case of 63−40 µm fraction. However, while the 40 µm fraction contains smaller particles, which also modify the composite surface facture, it does so without introducing much porosity. It is a known fact that materials with more complex surface facture often show increased hydrophobicity if the base material is hydrophobic itself. The composites studied were subjected to distilled water at 50 °C in order to check the effects of such conditions on the degree of hydrophobicity, mechanical properties and to assess the water tightness of the printed objects. After the samples treatment with water at 50 °C, the contact angle values increased considerably to above 113°. Higher water contact angles were measured for the composites containing diatom shells fraction of smaller particle sizes <40 mm. With an increasing content of the filler, the water contact angle decreased. The greatest increase in the WCA relative to that of the reference sample was obtained for the composites containing the diatomite size fraction 63−40 µm (by about 30.2°). A significant increase in WCA was caused by changes in the microstructure of the sample surface (additional surface structuration). Additionally, PLA is a semicrystalline polymer and there are phases that undergo hydrolysis faster (amorphous phase) and slower (crystalline phase, also known as spherulites). Therefore, during hydrolysis, different spots of the surface hydrolyze with different ratios, resulting in additional factorization. It is visible as the neat PLA also becomes more hydrophobic after the water treatment. The WCA of the composites is a superposition of two effects: a physical structural one and a chemical one on the surface. The physical effect is dominant for samples on the inhomogeneous surface. The use of greater particle size fraction of the filler and its greater content lead to a decrease in the degree of hydrophobicity, thanks to its hydrophilic properties. The samples containing the fraction <40 µm are characterized by higher WCA than those containing the fraction of particle sizes 40−63 µm, which is explained by the fact that smaller filler particles do not lead to the appearance of large hydrophilic defects on the sample surface that would decrease the degree of hydrophobicity. With an increasing concentration of the filler, WCA decreases as a greater amount of hydrophilic filler is present on the sample surface.

**Figure 7:**
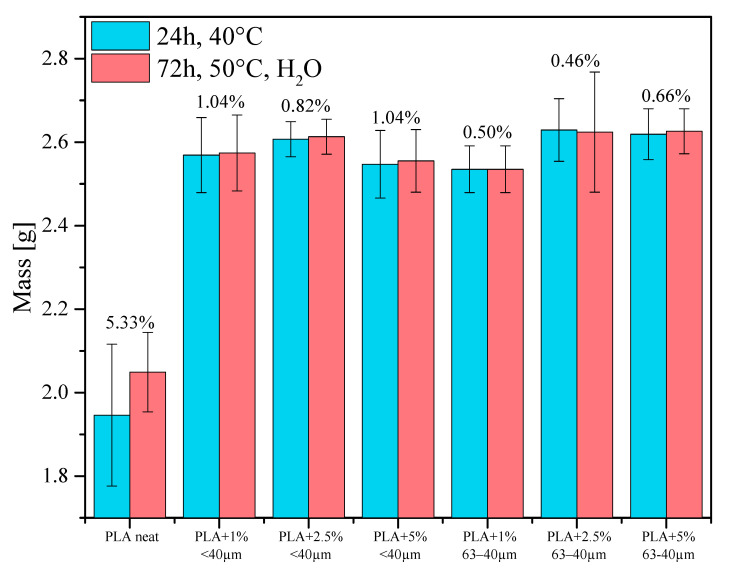
Masses of the as-obtained samples and the samples conditioned in distilled water at 50 °C for 72 h.

PLA subjected to chemical treatment in water undergoes hydrolysis. On the basis of measurements of the composites mass before and after the conditioning in water it was proven that water remains in the samples’ pores. The mass increase was the greatest for the samples of neat PLA. The samples containing diatomaceous earth were more resistant to water; their mass increased by almost 1.04%, while the mass increase of neat PLA was almost five times greater. While the water absorption by the samples studied was rather poor, their hydrophobic properties significantly increased. The hydrolysis of hydrophobic PLA led to a stronger binding of the polymer with polar filler, which caused an additional effect on the micro-structuration of the samples’ surfaces, leading to partial pore closing and an increase in the samples hydrophobicity (Figure 7).

### 3.6. XRD

**Figure 8:**
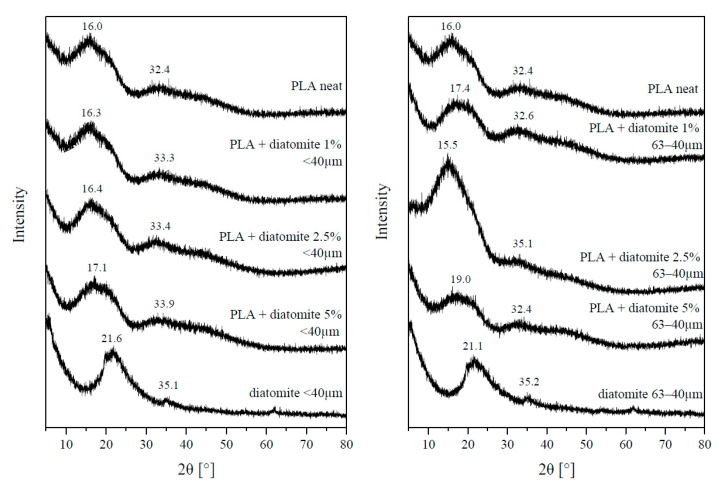
Diffractograms of PLA composites with diatom shells.

All the PLA/diatomite composites, as well as starting materials, neat PLA and diatomite of both diameter fractions, were subjected to XRD measurements (Figure 8). Due to the low crystallinity levels of the materials, the diffractograms present broad diffraction peaks instead of sharp reflexes. PLA shows two peaks, at. 2θ 16.0°, coming from (110) and (200) lattices, and a low-intensity 32.4°, coming from the (216) lattice. The observed crystalline phase is the most common α type [30]. Diatomite also shows two crystalline peaks at ~21° and ~35° (depending on the defects of the crystalline structure in the particular material grade and species of the diatoms the material is derived from). For the PLA/diatomite composites, it can be observed that although particular reflexes or peaks cannot be distinguished, the moderately intense 16.0° peak of PLA is broadened and its maximum is shifted to higher angles, which is a result of diatomite 21° peak contribution. This effect is stronger for 63−40 µm diatomite composites. Moreover, an interesting effect was observed for the 2.5% composite of 63−40 µm diatomite/PLA, where application of the filler resulted in improved polymer crystallinity, visible as the increase of peak intensity. The unexpected shift in the peak maximum, however, may be explained by the internal stress of the sample, residual from the composite processing (known also as the material processing memory). The effect, non-observable for the 5% composite of 63−40 µm diatomite, may be due to low control over material crystallization behavior, which was not a factor studied in this work.

### 3.7. Mechanical Properties

#### 3.7.1. Tensile Strength and Flexural Strength

**Figure 9:**
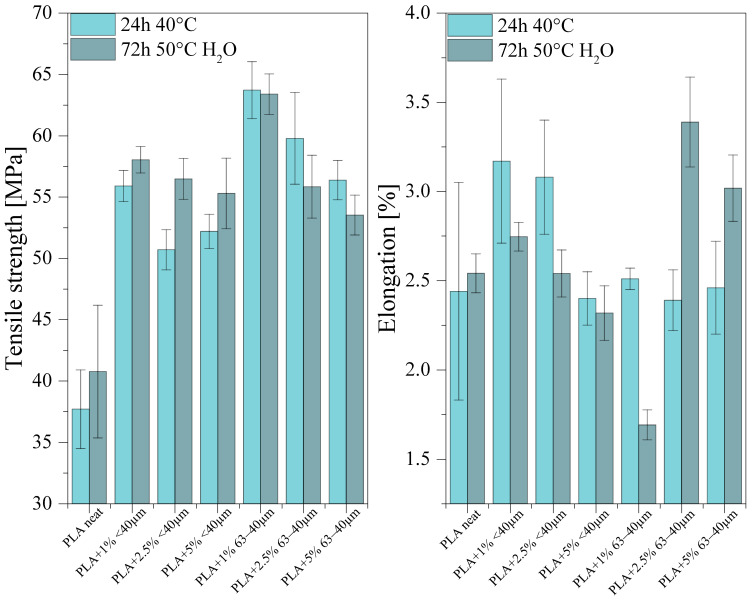
Results of tensile tests.

Neat PLA samples for tests, printed out by the FDM method, showed tensile strength of 15.5−72.2 MPa, elongation at rupture of 0.5−9.2 %, bending strength of 52–115.1 MPa and elasticity modulus of 2.39–4.93 GPa [31,32]. The samples of PLA composites with diatom shells printed out by the same method showed almost a twice as high tensile strength (maximum value of 63.7 ± 2 MPa) relative to the value for neat PLA (37.7 ± 3 MPa). The greatest mechanical strength was noted for the samples containing 1% of diatomaceous earth. Higher concentrations of the filler result in a decrease in the tensile strength, which is related to a too high number of discontinuities in the polymer. Elongation at rupture was at a similar level for all the samples, however, the samples containing 1% or 2.5% of the filler of particle size <40 µm showed a small increase in the value of this parameter, up to >3% (Figure 9). After examination of water absorbability at 50 °C, the tensile strength increased, both for neat PLA and for PLA composites with diatomaceous earth fraction <40 µm. The composite samples with the filler particle size fraction 63−40 µm revealed a subtle decrease in tensile strength. More pronounced differences were observed for elongation at rupture. The elongation of neat PLA samples remained basically the same, but for the composites containing 2.5% or 5% of the filler of grain size 40−63 µm, the values of this parameter increased to about 3.5 and 3.0, respectively, so the increase reached 20% to 40%. For all samples, high values of standard deviation were obtained, which is characteristic of the samples printed by FDM.

**Figure 10:**
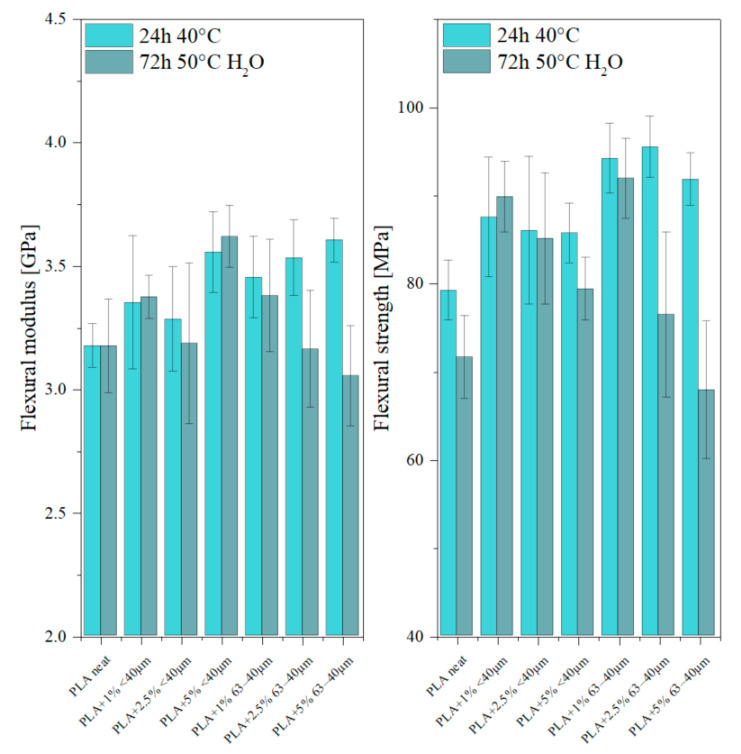
Results of bending tests.

The elasticity modulus in bending and bending strength of the samples were measured by the three-point bending flexural test at the rate of 2 mm/min, (Figure 10). The samples of composites showed higher elasticity modulus in bending and higher flexural strength than neat PLA.

The value of elasticity modulus significantly depends on the content of the filler in the composites; it increases with increasing filler concentration. The addition of diatomaceous earth also increases the flexural strength of the samples, which is more pronounced for the samples containing the particle size fraction 40−63 µm, but the differences between the concentrations are within the standard deviation. After the studies of water absorbability, the mechanical strength parameters decreased for the majority of samples. While water absorption by the composite does not induce changes in the final mass of the samples appreciably (Figure 7), it modifies the structure of the samples and thus affects their mechanical properties. The considerable deterioration of mechanical strength parameters is explained by a decrease in the average molecular mass and degree of crystallinity caused by the polymer hydrolysis. The loss of mechanical properties correlated with the increasing loadings of the fillers, especially visible for 63−40 µm fraction, may be due to hydrophilic properties of the diatomite component, increasing water penetration through the sample.

#### 3.7.2. Impact Strength and Hardness

The impact strength of neat PLA varies in the range 13.3 ± 4.4 kJ/m^2^ (Table 5). For all samples of the composites, the values of Kc are higher and vary in the range 18.5−21.8 kJ/m^2^. The hardness of the composites in the Shore D scale takes values from the range 79−83°, while the PLA hardness is close to 83, which is consistent with the literature data. After the exposition of the samples to water at 50 °C, the hardness of all the samples decreased slightly, which can be linked to the hydrolytic depolymerization of PLA. After treatment with water at 50 °C, the impact strength of neat PLA and PLA +1% diatomite <40 µm significantly decreased, by 83% and 65%, respectively, which was attributed to the degradation of PLA in water. For the other samples, the impact strength increased by from 4% to 20%. With increasing content of the filler, the effect of diatomite in the mechanical properties of the composite begins to dominate the influence of PLA degradation in water; moreover, the interaction between PLA and the filler increases. Thus, the samples of composites are more resilient and less brittle.

### 3.8. Rheology

**Figure 11:**
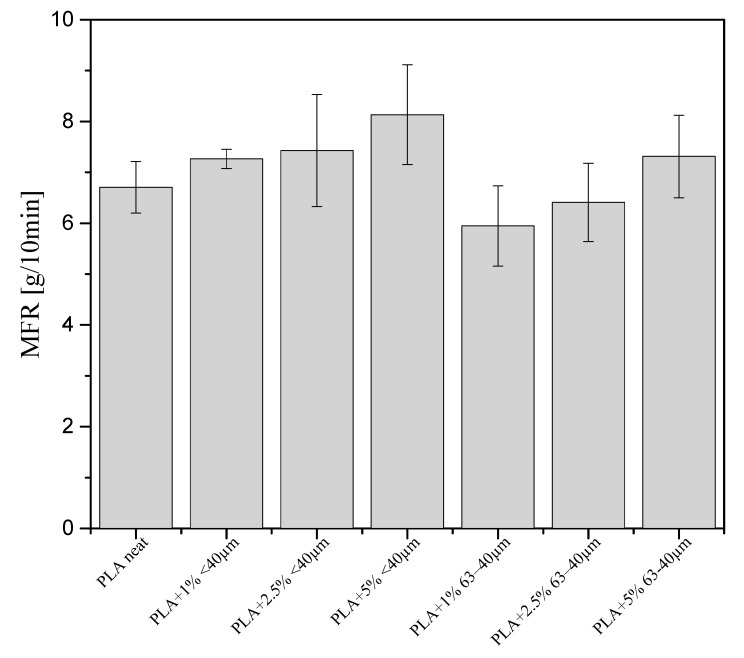
Results of flow coefficient measurements (tests carried out at 190 °C).

The melt flow index (MFR) of pure PLA at 190 °C is 6.705 g/10 min (Figure 11). For the PLA compositions with diatomaceous earth of grain size <40 µm the MFR value increased with increasing concentrations of the filler. The composite samples containing the filler fraction of 63−40 µm are characterized by smaller MFR value, although still MFR increases with increasing filler concentration. A higher MFR means that a given material can be easier processed and used for different applications. For the composites with the filler fraction of 63−40 µm, this parameter takes lower values as larger grains may cause greater turbulences in the polymer stream so the friction increases and MFR decreases.

**Figure 12:**
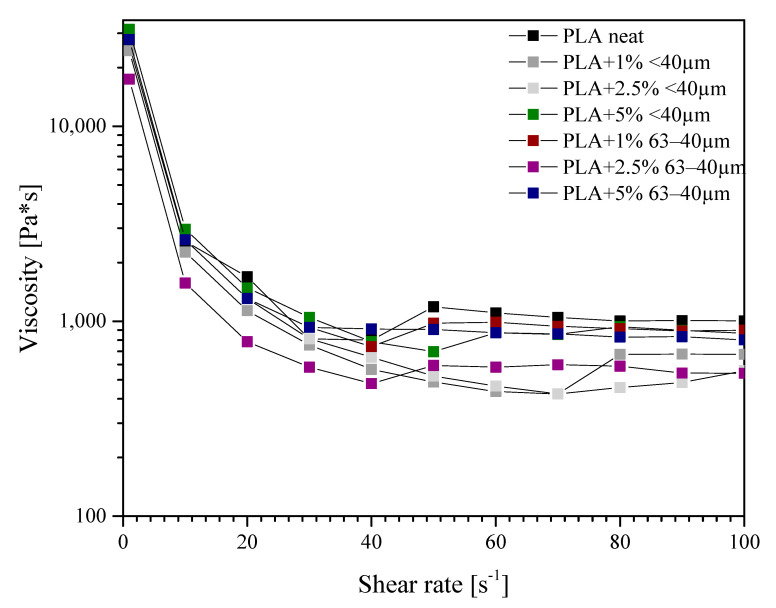
Viscosity measurement results.

A capillary rheometer was used to determine the relation between shear rate and viscosity (Figure 12). At the lowest shear rate, the viscosities of all compositions are greater than that of pure PLA, as a consequence of the presence of the filler. The highest increase in viscosity was noted for the sample containing 5% of the filler. With an increasing shear rate, the values of viscosity decrease, which is known as the shear thinning. Some of the samples, mainly containing 63−40 μm diatomite fraction and also 5% of 40 μm fraction, revealed an increase in viscosity near the shear rate of 50 1/s, attributed to the onset of composition turbulent flow.

### 3.9. Density Measurements

**Figure 13:**
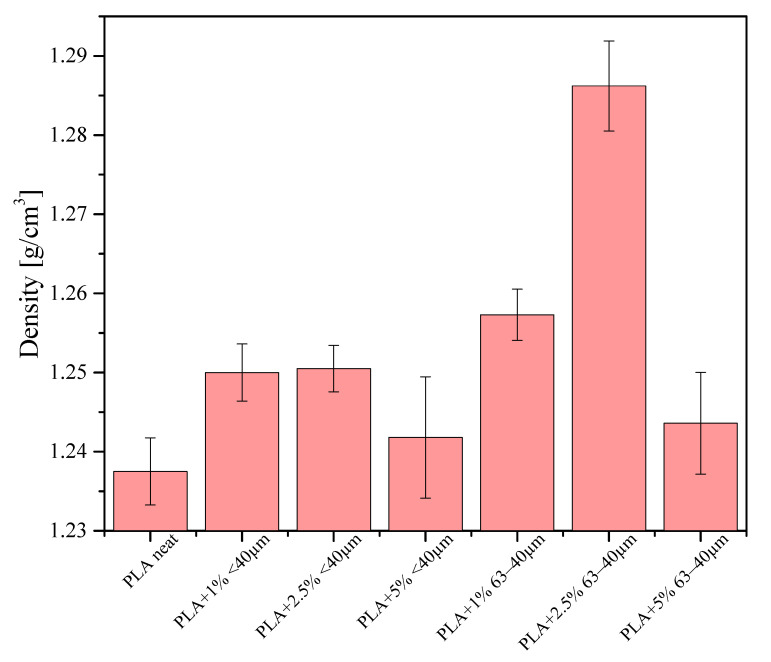
Densities of the extruded samples.

Densities of all samples were measured by the hydrostatic method and the results are given in Figure 13. Measurements were performed for the samples of 1 cm in length. The lowest value of density was found for neat PLA (~1.24 g/cm^3^). For the composites, with an increasing concentration of the filler, the density slightly increased up to 2.5% of the filler loading and at 5% loading, the density was abruptly reduced.

**Figure 14:**
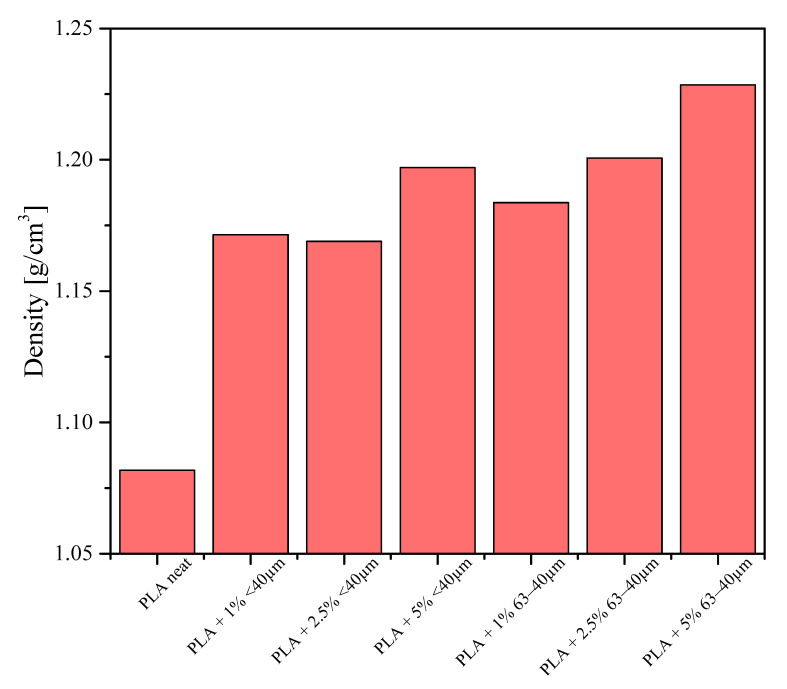
Densities of the 3D-printed samples for bending tests.

The greatest density was obtained for the sample containing 2.5% of the 63−40 μm fraction. The experimentally determined neat PLA density is in agreement with the literature value [33]. In parallel, densities of the samples printed out for bending tests were measured and the results are given in Figure 14. Their densities were calculated on the basis of measurements of their thicknesses, lengths, widths and masses. The samples studied were printed out on a 3D printer with 100% infill ratio. The samples of composites were found to have much higher density than that of pure PLA, which means that even a small addition of diatomite results in denser packing of the composition during printing and that the theoretical 100% infill ratio is not actually the most effective material packing to obtain. Irrespective of the mesh fraction of the filler, the sample containing 5% of the filler showed the highest density. These samples caused considerable problems during printing, e.g., blocking of the extruder, detaching of the sample from the printer table, in particular the samples containing the filler fraction of 63−40 µm.

## 4. Conclusions

The composites obtained by the modification of polylactide (PLA) with diatomaceous earth were characterized by higher thermal stability and a greater degree of hydrophobicity than neat PLA. Moreover, the composites were observed to show a higher WCA after treatment with water and its reduced absorbability (small mass change) than neat PLA. As to the mechanical strength parameters, the measurements for the composites revealed an increase in tensile strength and an improved elongation at rupture in some cases. Additionally, the samples were characterized by improved flexural durability. Modification of polylactide with diatomaceous earth results in increasing the melt flow in proportion to the concentration of the modifier. The use of different mesh fractions of the filler is reflected in different mechanical and rheological parameters of the composites, as well as in the density of the filaments. The composites obtained with the diatomite mesh fraction <40 µm show higher hydrophobicity, and after conditioning in water, higher melt flow index and higher values of most mechanical strength parameters. However, those obtained with the diatomite mesh fraction of 63−40 µm show greater thermal stability, greater tensile strength and the filaments made of such composites show greater densities.

The obtained composite filaments may be viewed as an attractive alternative to the ones currently available commercially for 3D printing and made from neat PLA, offering improved mechanical properties, a cut back on the PLA used for their manufacturing and being made solely from non-toxic, bio-friendly and biodegradable materials. The impact of the diatomite as a filler for PLA on its biodegradation kinetics and pathways will be the subject of further studies, as it is substantial from the perspective of an environmental sustainability-oriented development of new materials.

## Figures and Tables

**Table 1:** Process parameters for sample printing.

Layer height	0.18 mm
Top layer height	0.27 mm
Shells	2
Top and bottom layers number	3
Bottom layers number	3
Infill density	100%
Infill pattern	Hexagonal
Printing speed	60 mm/s
Idle speed	80 mm/s
Extruder temp.	220 °C

**Table 2:** Results of thermogravimetric analysis.

	1% Mass Loss [°C]	Onset Temperature. [°C]	Temperature at the Maximum Rate of Mass Loss [°C]	Dry Mass Left after Pyrolysis [%]
Neat PLA	305.8	348.3	376.0	0.00
PLA + 1% diatomite < 40 µm	292.9	354.1	366.7	0.21
PLA + 2.5% diatomite < 40 µm	311.3	352.2	373.8	1.73
PLA + 5% diatomite < 40 µm	301.2	350.5	372.3	3.25
PLA + 1% diatomite 63–40 µm	312.1	353.2	373.5	0.60
PLA + 2.5% diatomite 63–40 µm	313.0	352.4	375.6	2.01
PLA + 5% diatomite 63–40 µm	311.4	351.4	372.5	3.74
Diatomite < 40 µm powder	66.9	194.5	234.8	86.09
Diatomite 63–40 µm powder	74.4	145.5	228.8	86.90

**Table 3:** Analysis of DSC results for the composite samples.

	T_g_ [°C]		T_c__c_ [°C]		T_m_ [°C]	
	Granulate	Print	Granulate	Print	Granulate	Print
PLA natural	59.9	57.7	126.4	124.6	153.3	151.5
PLA + 1% diatomite < 40 µm	57.0	57.6	121.6	123.1	152.5	152.8
PLA + 2.5% diatomite < 40 µm	58.2	59.6	119.5	119.1	153.3	150.5
PLA + 5% diatomite < 40 µm	57.3	58.3	116.3	117.8	151.5	151.6
PLA + 1% diatomite 63–40 µm	57.2	59.4	122.9	124.1	152.5	153.9
PLA + 2.5% diatomite 63–40µm	57.5	55.0	117.0	120.9	152.6	153.1
PLA + 5% diatomite 63–40 µm	58.0	55.1	118.6	118.7	152.0	152.5

**Table 4:** Water contact angle [°].

		Contact Angle [°]			
Storage Condition	Fraction	Neat PLA	PLA + 1% Diatomite	PLA + 2.5% Diatomite	PLA + 5% Diatomite
24 h, 40 °C	<40 µm	93.5	105.2	104.9	105.3
	63−40 µm		86.6	87.6	83.1
72 h, 50 °C, H_2_ O	<40 µm	112.9	123.8	115.2	113.8
	63−40 µm		117.4	116.3	114.3

**Table 5:** Impact test results and shore hardness.

	Impact Strength [kJ/m^2^] (24 h, 40 °C)	Impact Strength [kJ/m^2^] (72 h, 50°C, H_2_O)	Hardness Shore D [°] (24 h, 40°C)	Hardness Shore D [°] (72 h, 50°C, H_2_O)
Neat PLA	13.35 ± 4.42	2.22 ± 0.52	82 ± 2	77 ± 3
PLA + 1% diatomite < 40 µm	20.90 ± 4.39	7.37 ± 2.14	81 ± 1	79 ± 1
PLA + 2.5% diatomite < 40 µm	20.52 ± 5.78	24.58 ± 4.14	80 ± 1	78 ± 2
PLA + 5% diatomite < 40 µm	18.51 ± 3.47	19.3 ± 6.97	83 ± 2	79 ± 1
PLA + 1% diatomite 63–40 µm	21.81 ± 4.42	27.19 ± 2.64	81 ± 3	75 ± 2
PLA + 2.5% diatomite 63–40 µm	20.24 ± 5.10	21.66 ± 4.60	80 ± 1	78 ± 1
PLA + 5% diatomite 63–40 µm	20.54 ± 3.14	25.11 ± 1.38	79 ± 1	77 ± 1
